# Three Genes Involved in Different Signaling Pathways, *carS*, *wcoA*, and *acyA*, Participate in the Regulation of Fusarin Biosynthesis in *Fusarium fujikuroi*

**DOI:** 10.3390/jof10030203

**Published:** 2024-03-08

**Authors:** Violeta Díaz-Sánchez, Marta Castrillo, Jorge García-Martínez, Javier Avalos, M. Carmen Limón

**Affiliations:** Department of Genetics, Faculty of Biology, University of Seville, 41012 Seville, Spain; violeta.diaz@dcaf.uhu.es (V.D.-S.); avalos@us.es (J.A.)

**Keywords:** Fusarins, photoreceptor, Fus1 polyketide synthase, White Collar, adenylate cyclase, CarS

## Abstract

The phytopathogenic fungus *Fusarium fujikuroi* has a rich secondary metabolism which includes the synthesis of very different metabolites in response to diverse environmental cues, such as light or nitrogen. Here, we focused our attention on fusarins, a class of mycotoxins whose synthesis is downregulated by nitrogen starvation. Previous data showed that mutants of genes involved in carotenoid regulation (*carS*, encoding a RING finger protein repressor), light detection (*wcoA*, White Collar photoreceptor), and cAMP signaling (AcyA, adenylate cyclase) affect the synthesis of different metabolites. We studied the effect of these mutations on fusarin production and the expression of the *fus1* gene, which encodes the key polyketide synthase of the pathway. We found that the three proteins are positive regulators of fusarin synthesis, especially WcoA and AcyA, linking light regulation to cAMP signaling. Genes for two other photoreceptors, the cryptochrome CryD and the Vivid flavoprotein VvdA, were not involved in fusarin regulation. In most cases, there was a correspondence between fusarin production and *fus1* mRNA, indicating that regulation is mainly exerted at the transcriptional level. We conclude that fusarin synthesis is subject to a complex control involving regulators from different signaling pathways.

## 1. Introduction

*Fusarium fujikuroi* is a fungal pathogen responsible for bakanae disease in rice whose most characteristic symptom is the elongation of infected plants, and it is responsible for economic losses due to low seed yields. The elongation is caused by gibberellins produced by *F. fujikuroi*, including GA_3_ (gibberellic acid), GA_4_, and GA_7_; however, in infected plants, other secondary metabolites such as bikaverin, *O*-methylfusarubin, fusaric acid, apicidin F, beauvericin, and fusarins were detected by high-performance liquid chromatography (HPLC) [1]. In recent years, new secondary metabolite gene clusters were discovered in *F. fujikuroi* thanks to genome sequencing, and subsequently, the activation of silent ones led to the identification of those for apicidin F, beauvericin, fujikurins, gibbepyrone, trichosetin, and N-dimethylallyltryptophan [2,3,4,5,6,7,8].

**Figure 1:**
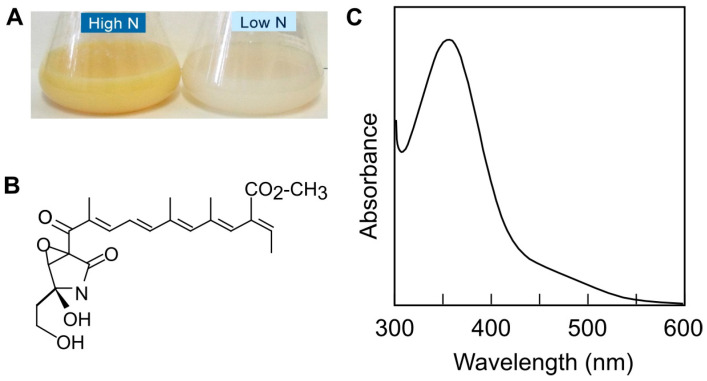
Fusarin production and detection. (**A**) Cultures of *F. fujikuroi* wild-type strain FKMC 1995 grown in DG medium with 20 mM (high N) or 4.2 mM of asparagine (low N). (**B**) Chemical structure of fusarin C. (**C**) Absorption spectrum of fusarins with a characteristic peak at 350 nm. Presence of fusarins in high-N culture in (**A**) is indicated by yellow color due to their absorption at 400–550 nm.

The focus of this work is on fusarins, mutagenic polyketides produced by different *Fusarium* species that include *F. fujikuroi* (Figure 1), *F. verticillioides*, *F. graminearum*, certain *F. proliferatum*, and many *Fusarium* isolates [9]; however, they were not detected in *F. oxysporum* [1,10,11]. Fusarin production is not exclusive to the *Fusarium* group and has also been found in *Metarhizium anisopliae* [12].

The polyketide synthase (PKS) gene involved in fusarin biosynthesis, *PKS10*, has been identified in the *Fusarium* species *F. moniliforme*, *F. venenatum* [13], *F. graminearum* [14], and *F. fujikuroi* [15]. This 12-kb gene codes for a PKS-non-ribosomal peptide synthetase (NRPS) hybrid enzyme. In *F. fujikuroi*, the *PKS10* gene was named *fus1* (also *fusA* [15]), and it is clustered with other genes, as in *F. verticillioides* [7,16]. This complete cluster is missing in some *Fusarium* species such as *F. oxysporum*, *F. mangiferae*, some *F. proliferatum*, and most of the *F. incarnatum–equiseti* species complex [7,17]. The fusarin cluster contains nine genes named *fus1* to *fus9*. The essential genes for fusarin biosynthesis are *fus1*, *fus2*, *fus 8*, and *fus9*, which code for a PKS-NRPS, a putative α/β hydrolase, a cytochrome P450, and a methyltransferase, respectively. The connection of the other genes of the cluster with fusarin production is unknown except for *fus6*, which codes for a transporter from the major facilitator superfamily that could be involved in fusarin secretion.

As secondary metabolites, the synthesis of fusarins in *F. fujikuroi* responds to the presence of diverse environmental signals, including nitrogen availability (Figure 1), pH, and light [15,18]. The *fus* genes are expressed under acidic pH conditions; however, these genes are not regulated by the PacC transcription factor as their expression is not altered in *pacC* mutants. On the other hand, the fusarin gene cluster lacks a specific regulator gene [18], but its expression is presumably connected to different regulatory networks involving global regulators, some of which are investigated in this work.

The amount of nitrogen and the quality of the nitrogen source are often important factors affecting the regulation of secondary metabolite production in fungi. In *F. fujikuroi*, many of the studied secondary metabolites, such as gibberellins, bikaverin, fusarubins, and carotenoids, are produced under nitrogen starvation while others that include fusarins, fusaric acid, apicidin F, and gibepyrone A are induced by nitrogen. All the genes of the *fus* cluster are induced in the presence of a high concentration of a good-quality nitrogen source, e.g., glutamine, and the genes have also been described to be regulated by glutamine synthase [15,18,19]. However, fusarin biosynthesis is independent of proteins involved in nitrogen regulation, such as AreA, AreB, and MeaB [19,20], but it is affected by NmrA [20]. Moreover, the velvet protein Vel1 has also a role in fusarin regulation, as shown by the reduction in fusarin production in *vel1* deletion mutants [21].

Light is an environmental signal that regulates numerous aspects of fungal metabolism [22]. Light is detected by photoreceptor proteins, which transmit the signal to trigger responses at the gene expression level. The most studied photoreceptor system in fungi is the White-Collar complex, consisting of two proteins, WC1 and WC2 [23]. Of these, the photoreceptor function is carried out by WC1, which contains a LOV (Light, Oxygen, Voltage) domain that binds a flavin molecule as a chromophore. The genome of *F. fujikuroi* contains genes for several photoreceptors [24], including an orthologue of WC1 called WcoA. A phenotypic analysis of *wcoA*-disruption mutants showed that they are affected in the production of different secondary metabolites [25]. A subsequent transcriptomic analysis revealed that WcoA is a central regulator in *F. fujikuroi* and is involved in most transcriptional responses to light and in the regulation of hundreds of genes in the dark [26]. The mutation alters the expression patterns of numerous genes involved in secondary metabolism, including the fusarin cluster genes, which are mostly downregulated in the *wcoA* mutant.

The metabolites best known to be regulated by light are carotenoids [27]. In other fungi, such as *N. crassa*, the light regulation of these compounds is mediated exclusively by the WC complex [23], but *F. fujikuroi wcoA* mutants conserve their photoinduction, and so other photoreceptors are involved. Two of them, the cryptochrome CryD and the flavoprotein VvdA, have been investigated [28,29], and their mutations alter the pattern of the photoinduction of carotenogenesis [30]. Furthermore, in the case of CryD, its loss affects the production of other secondary metabolites, such as bikaverin [28]. It is not known whether these photoreceptors may be involved in the regulatory networks that participate in the control of fusarin synthesis.

Carotenoid synthesis is repressed by the CarS protein [27]. Mutations in the *carS* gene provoke a deregulation of genes involved in carotenogenesis and give rise to the overproduction of carotenoids. In addition, the mutants are affected in the synthesis of other metabolites, such as gibberellins and bikaverins. Recently, it was shown that the *carS* mutation brings about changes in the expression of hundreds of genes in addition to those of the carotenoid pathway, many of them also regulated by light [31]. CarS is a protein with two RING finger domains, found in E3-type ubiquitin ligases, and a LON domain. Considering these characteristics, the action of the regulator CarS is presumably carried out by modifying the activity of target proteins.

Adenylate cyclase is responsible for cAMP synthesis and thus plays a key role in the cAMP-PKS signaling pathway, one of the most investigated global regulatory pathways in *Fusarium* [32]. Mutants lacking adenylate cyclase activity show various phenotypic alterations in different *Fusarium* species, including pathogenicity, development, or stress resistance [33,34,35]. In this pathway, cAMP activates protein kinase activity which phosphorylates different substrates, such as the regulatory protein Sge1 [32], which is involved in the regulation of secondary metabolism [36], and mutations in adenylyl cyclase frequently provoke changes in the production of different metabolites. In *F. proliferatum* and *F. verticillioides*, adenylyl cyclase mutants are affected in bikaverin biosynthesis [33,37], and in *F. graminearum*, a gain-of-function mutation in adenylyl cyclase leads to the overproduction of deoxynivalenol [38]. In *F. fujikuroi*, the cAMP-PKS signaling pathway is involved in secondary metabolism [39], and adenylyl cyclase mutants are affected in the production of different metabolites including fusarubin and gibberellins [39,40].

Unlike other metabolites, such as bikaverins or gibberellins, the regulation of fusarin synthesis has not received much attention in *Fusarium*. We have taken advantage of the availability of mutants in the *carS*, *wcoA*, and *acyA* genes, which encode proteins involved in different signaling pathways. Previous data showed that they are connected in different ways to the nitrogen regulation of secondary metabolism, as found for CarS [41,42], WcoA [26], and AcyA [43]. In this work, we investigated the involvement of these proteins in the production of fusarins and in the transcriptional regulation of their pathway, the latter based on their effect on *fus1* gene mRNA levels.

## 2. Materials and Methods

### 2.1. Strains and Culture Conditions

*Fusarium fujikuroi* FKMC 1995 wild type was obtained from the Kansas State University Collection (Manhattan, KS, USA). All mutant strains were previously obtained from FKMC 1995 and are described in Table 1. All the strains were kept in the Genetics Department Fungal Collection, University of Seville, Seville, Spain.

Unless otherwise stated, strains were grown in DGasn minimal medium, that is, DG medium [31] with L-asparagine instead of NaNO_3_ as a nitrogen source. To ensure the resistant character of the transformants with gene deletion or disruption with the hygromycin resistance cassette, the medium was supplemented with 50 mg/mL of hygromycin.

For analyses of fusarin production, the strains were grown in 500 mL Erlenmeyer flasks with 250 mL of DGasn at 30 °C in the dark or under illumination (3 W/m^2^ of white light, approximately 180 lux, provided by four OSRAM L, 18W/840 LUMLUX fluorescent lamps (OSRAM, Munich, Germany). The flasks were inoculated with 10^6^ conidia and incubated for 7 days on a rotary shaker at 150 rpm. To study the effect of nitrogen concentration, L-asparagine was used at two concentrations: 20 mM (high-N medium) and 4.2 mM (low-N medium).

Mycelia from the different experiments were filtered using a vacuum system, and the mycelia were frozen in liquid nitrogen and kept at −80 °C until use. For qRT-PCR, RNA was isolated from mycelia grown in the same conditions used for fusarin measurements.

A former RNA-seq study [31] was based in cultures obtained with 10^6^ conidia of *F. fujikuroi* IMI 58289 wild type and an SG39 *carS* mutant inoculated in 500 mL Erlenmeyer flasks containing 100 mL of DG medium. The cultures were grown protected from light for three days at 30 °C and 150 rpm. Under a red safelight, 25 mL samples of the cultures were transferred to 8.9-cm Petri dishes. Half of the dishes were illuminated with white light for 1 h, and the rest were incubated in a carboard box in the dark. The mycelia from the different experiments were filtered using a vacuum system, and the mycelia were frozen in liquid nitrogen and kept at –80 °C until use.

### 2.2. Fusarin Analysis

Fusarins were extracted from both mycelia and culture filtrates as described [15]. Lyophilized mycelia were ground with sea sand (Panreac Química SAU, Barcelona, Spain) in methanol in a Fast-Prep-24 homogenizer (MP Biomedicals, Illkirch, France). In the case of filtrates, 3 mL samples of medium were extracted twice with chloroform and resuspended in 0.2 mL of methanol after chloroform evaporation in a Concentrator Plus vacuum centrifuge (Eppendorf, Hamburg, Germany). The extracts were concentrated, and the amount of fusarins was determined at 350 nm using a UV/Vis spectrophotometer Beckman DU 640 (Beckman Coulter, Brea, CA, USA) as described [15].

### 2.3. Gene Expression Analysis

For the isolation of total RNA, the mycelia were lysed in a Fast-Prep^®^-24 homogenizer prior to the use of the RNeasy Plant Mini kit (Qiagen, Hilden, Germany). The total RNA concentration was estimated with a Nanodrop ND-1000 spectrophotometer (NanoDrop Technologies, Wilmington, DE, USA). Real-time qRT-PCR expression analyses were performed on total RNA samples as template as described [15], using a 7500 Real Time PCR System (Applied Biosystems, Waltham, MA, USA). Primer sets for the qRT-PCRs were designed using Primer Express^TM^ v2.0.0 software (Applied Biosystems). RTfusA-1F and RTfusA-1R primers (5′-TGATATGTCGCTTACGCAGATG-3′ and 5′-CTCACTGGATGCAACGATCAG-3′) were used to quantify the *fus1* gene coding for the PKS. The primers tub-2F and tub-2R (5′-CGGTGCTGGAAACAACTG-3′/5′-CGAGGACCTGGTCGACAAGT-3′) specific for the β-tubulin gene were used as controls for constitutive expression. Relative gene expression was calculated using the 2^ΔΔCT^ method with Sequence Detection Software v1.2.2 (Applied Biosystems). Samples from two independent experiments were assayed in duplicate to ensure statistical accuracy.

### 2.4. Cloning and Sequencing of Mutant carS Alleles

Alleles were amplified from the genomic DNA of the SF114 and SF116 *carS* mutants via a PCR with primers Foxy 4F (5′-CTGGTGTATGAGATCTCTA-3′) and Foxy 3R (5′-CGAGAGATAGTAGGGCAAGC-3′), using the Expand High Fidelity DNA polymerase system. PCR fragments were cloned in a pGEM^®^-T plasmid (Promega, Madison, WI, USA) and sequenced in Stab Vida (Caparica, Portugal), using primers described in Supplementary Table S1.

### 2.5. RNA-Seq

The mRNA levels of the fusarin cluster genes were measured as transcripts per million (TPMs) using the Cuffdiff tool from previous RNA-seq sequencing data [31]. In that study, RNA was isolated with Trizol (Invitrogen, Paisley, UK), and samples were processed by Life Sequencing (Valencia, Spain) with Illumina protocol and sequenced on the Illumina HiSeq Platform (Illumina, Inc., San Diego, CA, USA) in a 50SE composition using the single-end methodology. Read counts for each gene were first normalized for its length and then for the sequencing depth to compare the proportion of reads that mapped to a gene in each sample.

### 2.6. Statistical Analysis

Fusarin and mRNA level data were statistically analyzed using a one-way analysis of variance (ANOVA) at the significance level of α = 0.05. A Tukey HSD post hoc test was used to determine the differences between the groups using the one-way ANOVA calculator and Tukey HSD from Statistics Kingdom through the server https://www.statskingdom.com/index.html (accessed on 2 March 2024). Variables with the same letters indicate that the differences among their means are not statistically significant.

## 3. Results

### 3.1. Effect of wcoA Mutation on Fusarin Production in F. fujikuroi

To obtain more insights into the role of WcoA in the regulation of fusarins, the production of these toxins was studied in two *wcoA* mutants, SF225 and SF226, under different culture conditions. Because fusarins are regulated by nitrogen availability, we cultured the *wcoA* mutants and a wild-type strain in media with 20 mM of asparagine (high-N medium) or 4.2 mM of asparagine (low-N medium). Fusarins were quantified both from mycelia and culture filtrates of the strains.

**Figure 2:**
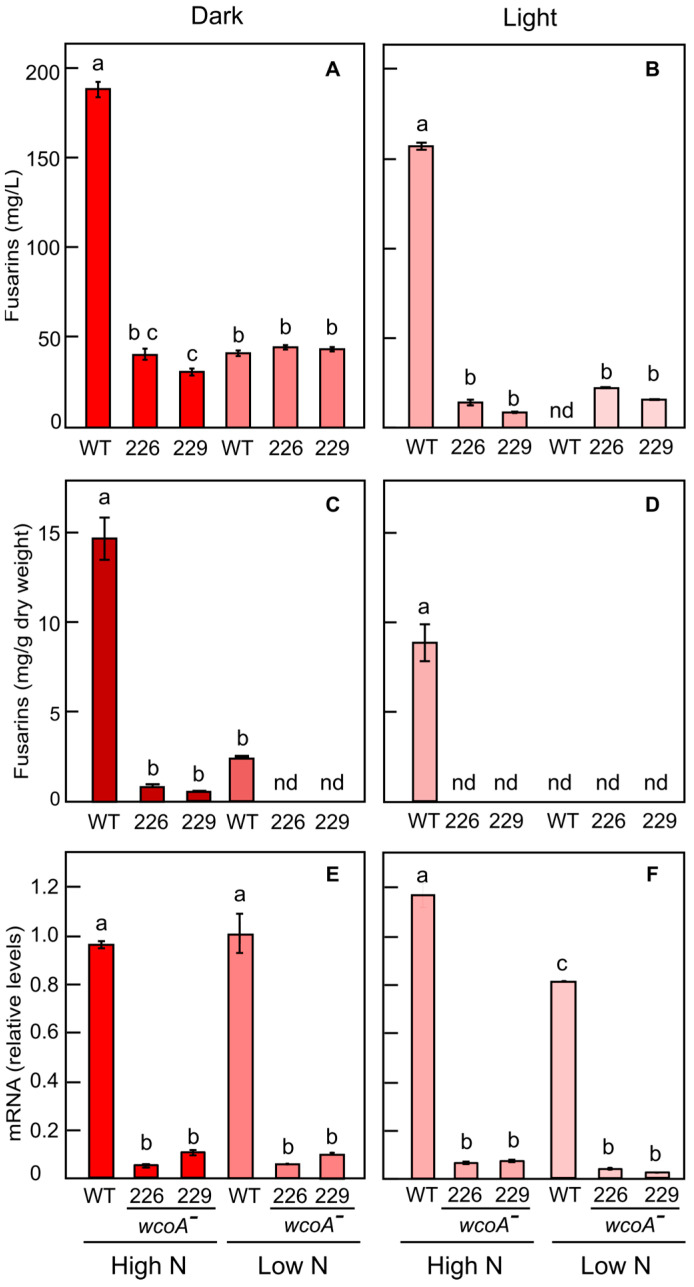
The effects of nitrogen concentration and illumination on fusarin production and *fus1* gene expression in a *F. fujikuroi* wild-type strain (WT) and the *wcoA*^−^ mutants SF226 and SF229. (**A**,**B**) Amount of fusarins secreted into the culture media. (**C**,**D**) Fusarins accumulated in the mycelia of the WT, SF226, and SF229 strains. (**E**,**F**) Effect of *wcoA* deletion on *fus1* mRNA levels in the same strains. (**A**,**C**,**E**) Cultures in darkness. (**B**,**D**,**F**) Cultures under illumination. The effect of *wcoA* mutation on *fus1* mRNA levels was measured by qPCR, using tubulin as an endogenous control. Mycelia were grown in liquid DGasn media with 20 mM (High N) or 4.2 mM of asparagine (Low N) at 150 rpm. Strains were incubated in darkness (left panels) or light (right panels) at 30 °C for 7 days. Filtrates and mycelia were taken from the same cultures. Fusarin data show average and standard deviations from two independent experiments. Expression data represent the average and standard deviation of 4 measurements from 2 independent experiments. Statistically significant differences are indicated with different letters according to the Tukey HSD test for a significance level of α = 0.05.

In darkness, the amount of fusarins secreted by the *wcoA* mutants in the high-nitrogen medium was reduced fourfold in comparison to those secreted by the wild type (Figure 2A), and an even stronger reduction was found in the mycelium (Figure 2C). As expected, fusarin production was reduced in the low-nitrogen medium. Under these conditions, the *wcoA* mutation did not affect the amount of fusarins secreted by the mutants compared to that of the wild type (Figure 2A), but a strong reduction in fusarin concentration was observed in the mycelia of the mutants (Figure 2C).

Under illumination, the results were similar, with a strong reduction in fusarin production by the *wcoA* mutants in the high-nitrogen medium, either secreted into the medium (Figure 2B) or accumulated in the mycelium (Figure 2D). In contrast, in the low-nitrogen medium, fusarin production was extremely low in the wild type and the *wcoA* mutants (Figure 2B,D), although an increase was observed in the filtrates of the mutants (Figure 2B) but not in the mycelium (Figure 2D). Overall, fusarin levels decreased in the presence of light in all strains.

Irrespective of nitrogen availability or the presence of light, the *wcoA* mutants had strong reductions in their mRNA levels of *fus1*, the gene encoding the polyketide synthetase responsible for fusarin biosynthesis (Figure 2E,F). This did not always correlate with the production of fusarins, indicating other mechanism of regulation at the posttranscriptional level. In conclusion, the regulator WcoA has an important role in the production of fusarins because a lack of WcoA caused a clear reduction under most of the conditions tested.

### 3.2. Effects of cryD and vvdA Deletion on Fusarin Production

**Figure 3:**
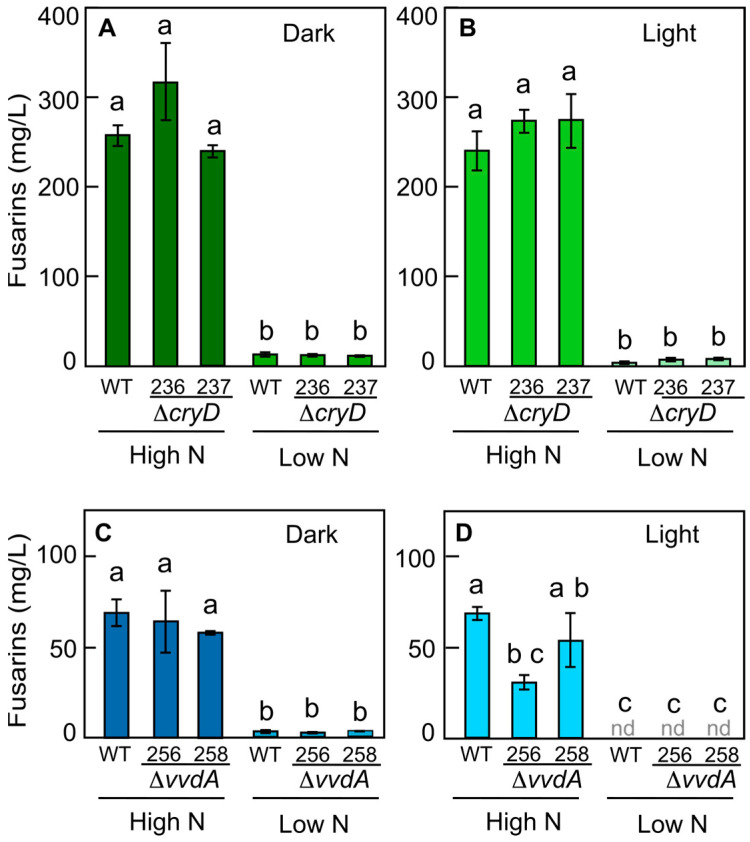
Amounts of fusarins secreted by a wild-type strain and Δ*cryD* and Δ*vvdA* mutants under different nitrogen concentrations and illumination conditions. (**A**,**B**) Amount of total fusarins secreted by the wild type (WT) and the Δ*cryD* mutants SG236 and SG237 into the medium. (**C**,**D**) Fusarins produced and secreted into culture medium by the WT and the Δ*vvdA* mutants SG256 and SG258. The strains were incubated for 7 days at 30 °C without (**A**,**C**) or with illumination (**B**,**D**). Statistical differences are indicated with different letters according to a Tukey HSD test for a significance level of α = 0.05.

Available mutants carrying deletions of the *cryD* and *vvdA* genes, coding for cryptochrome CryD and the small flavoprotein Vivid, respectively, were used to determine the potential roles of other photoreceptors in the regulation of fusarins. In the high-nitrogen medium, mutants lacking the *cryD* gene did not show significant differences in the amount of fusarins secreted into the culture medium compared to the wild type (Figure 3A,B), according to the Tukey HSD test. In the low-nitrogen medium, the mutants also behaved similarly to the wild-type strain, and the fusarin production of all the strains strongly decreased in comparison to the high-nitrogen conditions (Figure 3A,B). The amounts of fusarins were not statistically altered according to the Tukey HSD test when the cultures were illuminated, indicating that light did not affect the Δ*cryD* mutants (Figure 3B). The wild-type strain and the mutants lacking *vvdA* had similar production levels in the high-nitrogen medium in the dark, and in the light they showed very low amount of fusarins with no significant differences (Figure 3C,D).

### 3.3. Effect of carS Mutations on Fusarin Production

Fusarin production was studied in two *carS* mutants that accumulate high amounts of carotenoids, SF114 and SF116, derived from FKMC 1995 [42]. Both strains exhibit an increase in the mRNA levels of genes involved in the synthesis of neurosporaxhantin as *carB*, *carRA*, and *carD* [28,42] and have mutations in the *carS* gene affecting the RING finger domains. The SF114 *carS* allele has a transition (G287A) which causes a missense mutation affecting the last cysteine of RING finger 1 (Cys96Tyr). The need for four cysteines in this domain to be functional explains the *carS* mutant phenotype. On the other hand, the SF116 *carS* allele has a 7-bp deletion resulting in a frameshift that produces a shorter truncated protein of 94 amino acids that contains RING finger 1 but lacks RING finger 2 as well as the LON protease domain.

**Figure 4:**
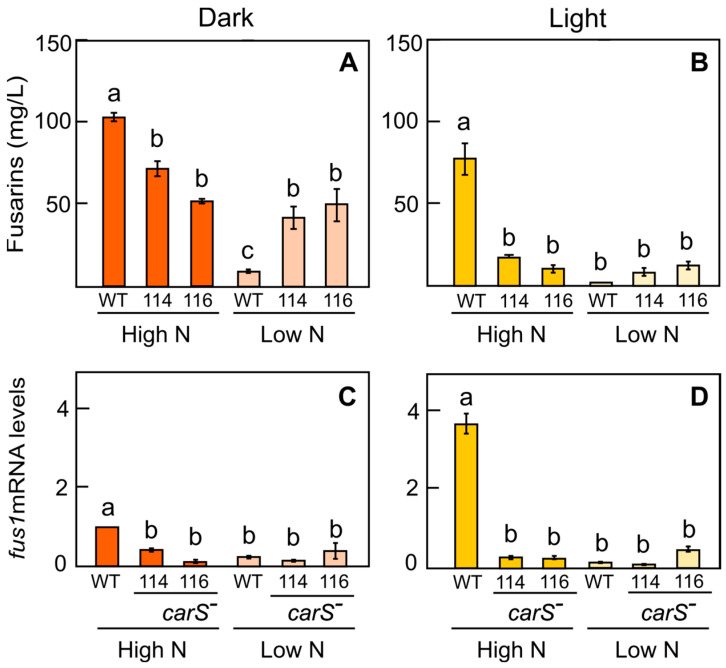
Fusarin production and *fus1* expression by the wild type and *carS* mutants SF114 and SF116, grown under different conditions of nitrogen and illumination. (**A**,**B**) Fusarins in the filtrates of 7-day-old cultures at 30 °C in minimal medium with high N (20 mM asparagine) and low N (4.2 mM asparagine) concentrations in the dark (left) and in the light (right). Data show mean and standard deviation values from two independent experiments. (**C**,**D**) Relative *fus1* mRNA amounts in mycelia from the cultures whose fusarin production levels are shown in the upper graphs. Data represent mean and standard deviation values from 4 measurements from 2 independent experiments. Statistically significant differences are indicated with different letters according to the Tukey HSD test for a significance level of α = 0.05.

The *carS* mutants exhibited similar alterations in fusarin biosynthesis. In the high-N medium, fusarin production was reduced significantly in them compared to the wild type (Figure 4A). However, production in the light in the high-N medium was drastically reduced in the *carS* mutants (Figure 4B). As expected from the nitrogen regulation of the pathway [15], in the low-N medium, fusarin production by the wild type was much lower, either in the dark or under light, than in the high-N medium (Figure 4A,B). However, there was a statistical increase in fusarins produced by the *carS* mutants in the low-nitrogen medium in the dark compared to the wild type (Figure 4A). The results were similar in the light, but in this case, the increase in the mutants did not reach the strict significance demanded by the statistical test. These data suggest that the *carS* mutants do not respond to the mechanism that represses the fusarin pathway under nitrogen starvation and that this deregulation is independent of light.

In either high- or low-N media, fusarin concentrations were lower in the illuminated cultures than in those grown in the dark (Figure 4A,B), a result also observed in the study of the *wcoA* mutation (Figure 2A–D). This could be explained by the degradation of fusarins by light, as described previously [15], although other regulatory factors, e.g., differences in mRNA translation, are not ruled out.

**Figure 5:**
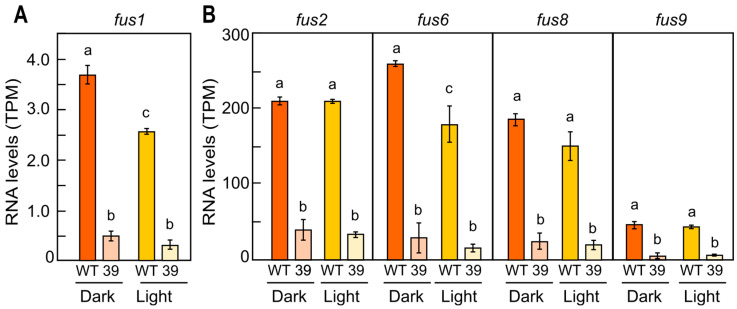
Transcript levels of fusarin genes in the wild type IMI 58289, and *carS* mutant SG39, isolated from this strain. (**A**) Transcripts per million (TPMs) of the *fus1* gene in both strains in the dark and in the light. (**B**) TPMs of a selection of fusarin cluster genes in the wild type (WT) and *carS* mutant in the dark and after illumination. Values obtained from the datasets of a former RNA-seq study on the influence of light and the CarS protein on the *F. fujikuroi* transcriptome [31]. Briefly, mycelia were grown for 3 days in flasks, and 25 mL samples were transferred to Petri dishes for illumination for 1 h or incubation in the dark. WT: wild type. 39: SG39 *carS* mutant. Statistically significant differences are indicated with different letters according to the Tukey HSD test for a significance level of α = 0.05.

The transcript levels of the *fus1* gene in the wild type and *carS* mutants grown in the high-N medium roughly correlated with their differences in fusarin production. Thus, the *fus1* mRNA levels were significantly lower in the *carS* mutants than in the wild type grown in both a high N concentration in the dark (Figure 4C) and under illumination (Figure 4D). These results were confirmed using the RNA-seq data of a *carS* mutant, SG39, and the wild-type strain of *F. fujikuroi* IMI58289 from which it was obtained illuminated for 1 h or maintained in the dark [31]. A similar down-regulation was obtained for other *fus* genes involved in the biosynthesis and export of fusarins in the *carS* mutant (Figure 5).

The transcription of *fus1* in the *carS* mutants did not show significant differences when cultured with a low nitrogen concentration in comparison to a high nitrogen concentration. Notably, *fus1* mRNA levels in the wild-type strain in the dark were significantly lower in the low-N medium compared to the high-N medium (Figure 4B), and this correlated with the observed differences in fusarin production (Figure 4A). The same correlation was observed when the wild-type mycelium was grown in the light (Figure 4B,D).

A third mutant of the *carS* gene available with the same genetic background, SF134, has a transition (G1133A) causing a missense mutation. The resulting amino acid change, Glu311Ala, affects the LON protease domain of the CarS protein while the RING finger domains remain intact [43]. This mutant allele resulted in alterations in fusarin production and *fus1* transcript levels (Supplementary Figure S1), reinforcing the participation of CarS in fusarin regulation. However, the alterations were not totally coincident with those exhibited by the mutants of the RING finger domains, SF114 and SF116, suggesting different regulatory functions of the LON domain.

### 3.4. Effect of ΔacyA Mutation and Nitrogen Concentration on Fusarin Production

**Figure 6:**
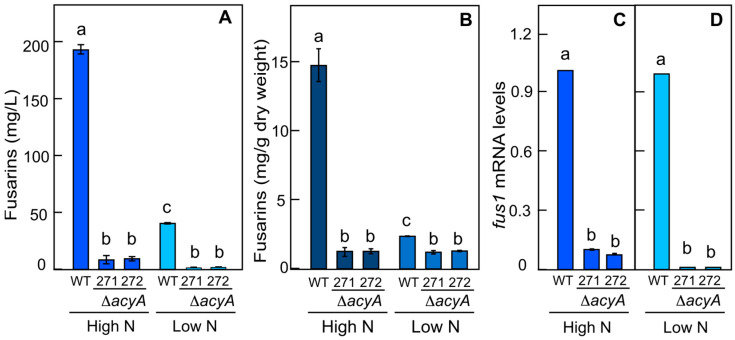
Effect of *acyA* deletion on fusarin production and *fus1* gene expression in cultures with high and low nitrogen concentrations. (**A**) Fusarin secreted into the culture media; (**B**) Fusarin accumulated in the mycelia. Cultures of the wild type (WT) and Δ*acyA* mutants SG271 and SG272 were grown at 30 °C for 7 days in the dark in medium with 20 mM asparagine (High N) or with 4.2 mM asparagine (Low N). Filtrates and mycelia corresponded to the same cultures. Data show the averages and standard deviations from two independent experiments. (**C**,**D**) Effect of nitrogen on *fus1* mRNA levels in the same strains grown in a high-N medium (**C**) or low-N medium (**D**). Data are average and standard deviation values of 4 measurements from 2 independent experiments. The same letters on the bars represent a lack of statistical significance in the differences among the strains on the conditions indicated, according to the Tukey HSD (*p* > 0.05).

Mutants lacking the *acyA* gene, SF271 and SF272, were incubated in high-N and low-N media. Regardless of the nitrogen concentration, the *acyA* mutants exhibited a drastic decrease in fusarin production in comparison to the wild type (Figure 6A,B). However, the fusarin levels in the mutants were not statistically different when compared both SF271 and SF272 grown in low-N and in high-N conditions (Figure 6A).

As described above (Figure 2 and Figure 4), the *fus1* mRNA levels were not affected by nitrogen concentration, confirming a posttranscriptional regulation of the fusarin pathway by this nutrient (Figure 6C,D). However, the number of *fus1* transcripts was strongly decreased in the *acyA* mutants compared to the wild type (Figure 6C,D). This involves cAMP signaling in the control of *fus1* transcription and strongly indicates that the lower level of fusarin production in the *acyA* mutants is due to a reduced expression of *fus1* and possibly also of other genes of the *fus* cluster.

## 4. Discussion

The biosynthetic pathways of secondary metabolites are often regulated by different environmental conditions, such as light or nutrient availability. Control mechanisms usually imply the participation of global regulators and eventually also pathway-specific transcription factors. The genes involved in the synthesis of each metabolite are typically clustered in the genome and may include a specific regulatory gene. The fusarin cluster is unusual because it lacks a specific regulatory gene and includes genes not needed for fusarin biosynthesis. Therefore, fusarin synthesis is probably controlled by the combined action of different global regulators.

The velvet protein Vel1 has also a role in fusarin regulation, as shown by the reduced fusarin production in the *vel1*-deletion mutants of *F. fujikuroi* [21] and by the lack of such production in the *FvVE1*-deletion mutants of *F. verticillioides* on cracked-corn cultures [44]. Moreover, decreased transcription of *pks10*/*fus1* was shown by loss-of-function mutants of the regulatory gene *laeA*, and the opposite was observed in *laeA*-overexpressing strains [45]. In the latter, the level of transcription depended on the amount of nitrogen present in the culture. The results obtained with the regulatory protein LaeA indicate that the *fus* cluster is also regulated at the epigenetic level. In fact, the loss of Kmt5, a H4k20 methyltransferase, provokes a strong increase in fusarin levels in *F. fujikuroi* cultures [46]. Fusarin biosynthesis was also upregulated in Δ*set1* mutants that are affected in the H3K4 trimethylation and downregulated in Δ*kmd5* mutants lacking the counterpart H3K4me3-specific demethylase [47].

Former data indicate the participation of other proteins in the regulation of fusarin biosynthesis in *Fusarium*. Thus, *fus* genes were not expressed in *gln1* mutants [18], indicating that glutamine synthetase plays an important regulatory role. An unexpected case was found in *F. verticillioides*, in which a genome-wide transcriptomic analysis of a mutant lacking the *FVEG_10494* gene coding for an aminotransferase showed that five genes of the fusarin gene cluster were induced [48].

Here, we report the involvement in fusarin regulation in *F. fujikuroi* of three additional regulatory proteins, WcoA, CarS, and AcyA. The participation of WcoA in the regulation of fusarin synthesis was expected since previous data on the effect of the mutation on the transcriptome found a patent decrease in mRNA levels in the *fus* cluster genes [26] and specifically in the four genes involved in the biosynthetic pathway, *fus1*, *fus2*, *fus8*, and *fus9* [18]. Our results confirm the role of WcoA not only at the level of *fus1* expression but also at the level of fusarin production. The strong decrease in fusarin production should be attributed to the lower mRNA levels for the pathway genes, although it could also be partially due to light-induced fusarin degradation during cultivation, as described for the wild type in a previous study [15]. WcoA is also a general regulator of other pathways of secondary metabolism since the *wcoA* mutation drastically reduces the expression of cluster genes for gibberellin, fusaric acid, and echisetin synthesis while producing the opposite effect on those for bikaverin synthesis. At least some of these pathways share that they are regulated by nitrogen, suggesting some linkage of WcoA with the AreA/AreB control system [49]. Interestingly, mutations in the *areA* or *areB* genes result in increased *fus1* expression in low-nitrogen conditions and decreased *fus1* expression in high-nitrogen conditions, whereas the *wcoA* mutants exhibit decreased *fus1* expression irrespective of nitrogen availability.

In the case of the *wcoA* analysis, fusarin concentrations were determined both internally and externally, which may provide information on fusarin secretion activity. The concentration of fusarins in the mycelium was at least 10-fold higher than in the culture medium. However, the internal concentration referred to the mycelial dry mass (mg/g) and it is therefore not directly comparable to the external values in mg/L. Indeed, the difference in concentrations would be much smaller or almost non-existent if the internal concentrations referred to the wet mass, in which 1 mg would be roughly equivalent to 1 mL. Therefore, considering that the external volume is much larger than the cell volume, most fusarins accumulate externally, which suggests the occurrence of a very active secretion mechanism.

Our data indicate that the role of WcoA in fusarin regulation is independent of its photoreceptor function since the mutation produces similar effects on *fus1* gene expression regardless of the presence of light. This fact highlights the dual role of WcoA as a regulator in the dark and as a photoreceptor responsible for most transcriptional responses to light in *Fusarium* [26]. Former data suggest a cooperative participation of WcoA, CryD, and VvdA in the regulation of carotenoid production by light [24]. The lack of effects of the *cryD* and *vvdA* mutations on fusarin synthesis is consistent with the effect of the *wcoA* gene mutation since both genes are poorly expressed in darkness and are strongly photoinduced via the WC system.

The RNA-seq results for the *wcoA* mutants showed a downregulation of the expression of genes of the *fus* cluster except for the *fus4* mRNA reads, which were upregulated [26]. This gene codes for a peptidase with no obvious relation to fusarin production. The numbers of mRNA readings for the genes involved in the biosynthesis of fusarins, *fus1*, *fus2*, *fus8*, and *fus9*, were lower in the *wcoA* mutants than in the wild type in RNA-seq analyses carried out with a high nitrogen concentration in the dark [26]. This is consistent with the lower amounts of fusarins secreted into the culture medium by the *wcoA* mutants than by the wild-type strain.

*F. fujikuroi* mutants lacking the photoreceptors CryD and VvdA were not affected in the synthesis of fusarins. However, previous work reported that Δ*cryD* mutants are affected in the production of other secondary metabolites, such as bikaverin and gibberellins, only when cultured under light [28]. Similarly, *vivid* mutants of *Podospora anserina* were shown to have repressed the sterigmatocystin gene cluster when cultured in the light but not in the dark [50]. The available data indicate that photoreceptors have different effects on the synthesis of different secondary metabolites and that the CryD and VvdA photoreceptors do not play an important role in fusarin biosynthesis, consistent with the not important effect of light on *fus1* transcription and fusarin production.

RNA-seq data revealed that the relevant genes of the *fus* cluster are downregulated both in the dark and under light in a *carS* mutant obtained from a different wild-type strain (Figure 5). This indicates that CarS has a role in the regulation of the biosynthesis of fusarins, probably as a repressor. CarS is a protein mainly associated with negative regulatory roles, in many cases acting on light-regulated genes [31]. In our experiments, we did not observe significant effects of light on fusarin production or *fus1* expression in the wild type. However, the higher amounts of fusarins found in the cultures of different *carS*^−^ mutants grown under nitrogen starvation suggest that CarS participates in fusarin regulation through the repression of synthesis in low-nitrogen conditions. Interestingly, the effects produced vary in the *carS*^−^ strains, each with a different *carS* mutant allele, being especially striking in the SF134 strain, which contains a mutation that affects the LON domain but keeps the two RING fingers intact. However, the mutants SF114 and SF116 contain only one active RING finger. This difference may be the reason why the SF134 mutant produced a similar amount of fusarins as the wild type when grown in a high-N medium in the dark. Both mutants lacking one functional RING finger had a similar reduction in *fus1* mRNA levels in the low-N medium. As a tentative hypothesis, the mutant lacking the LON domain could not be able to degrade a repressor involved in fusarin synthesis that acts in the absence of nitrogen. The phenotype of the three *carS* mutants on carotenoid synthesis is, however, similar [42], suggesting different mechanisms of action of the CarS protein on both pathways, possibly due to differences in the ability of each mutant CarS version to recognize the corresponding target protein. In support that CarS can act differently on diverse regulatory targets, in *S. cerevisiae*, it has been found that the ubiquitin ligase that acts in response to nitrogen starvation [51] also acts by regulating PHO pathway genes [52], indicating that certain ubiquitin ligases act in distinct signaling pathways.

Adenylate cyclase has an important role in the biosynthesis of secondary metabolites in *Fusarium* species. In *F. fujikuroi*, it was previously reported that *acyA* deletion led to decreases in carotenoid and gibberellin production [40], while it enhanced the production of reddish metabolites, a result also observed in *F. verticillioides* [33]. Thus, the cAMP signaling pathway may play upregulatory and downregulatory roles in different biosynthetic pathways. In *F. graminearum*, it is involved in the production of the mycotoxin deoxynivalenol (DON), since Δ*Fgac1* mutants are unable to produce this toxin [34]. Genes involved in cAMP-mediated regulation have also been investigated in other fungi. Mutants of *Trichoderma virens* lacking adenylate cyclase showed reductions in the synthesis of viridiol and other secondary metabolites [53]. In *Aspergillus flavus*, aflatoxin production is affected in *acyA* mutants [54].

In addition, other genes of the cAMP signaling pathway were studied in relation to secondary metabolites. In *Trichoderma atroviride*, the deletion of the G protein alfa subunit gene *tga1* also results in a reduction in 6-pentyl-α-pyrone (6-PP), supporting the role of this cAMP signaling pathway in the regulation of secondary metabolites in this species [55]. As further examples, *F. graminearum* mutants of the adenylate-binding protein (FgCAP1) also showed a decrease in DON production [56], and mutants of the Cyclase-Associated Protein (CAP) of *A. flavus* produced lower amounts of aflatoxin B [57].

## 5. Conclusions

Our data add three new regulators to the synthesis of fusarins in *Fusarium* species, the photoreceptor WcoA, the RING Finger protein CarS, and the adenylate cyclase AcyA, uncovering an increasing regulatory complexity for this pathway. These findings are in addition to previous reports on other regulatory proteins, such as GlnA, LaeA, and Vel1, and indicate that fusarin synthesis is subject to control by different signaling pathways that modulate the response to a diversity of environmental cues. These may well include those associated with their interaction with the plant in the infection process, which remains to be investigated. It is to be expected that new regulators remain to be discovered, and a future challenge will be to determine the connections between them that determine their coordinated action.

## Figures and Tables

**Table 1:** *F. fujikuroi* mutant strains used in this work.

Strains	Affected Gene	Protein Function	Mutation Procedure	Reference
SF225 and SF226	*wcoA*	WC ^1^ flavin photoreceptor	Targeted gene disruption	[25]
SF236 and SF237	*cryD*	DASH ^2^ cryptochrome	Gene deletion	[29]
SF256 and SF258	*vvdA*	Flavin photoreceptor	Gene deletion	[30]
SF271 and SF272	*acyA*	Adenylate cyclase	Gene deletion	[43]
SF114, SF136, and SF134	*carS*	RING ^3^ finger protein	NTG ^4^-induced mutagenesis	[44]

^1^ WC: White Collar. ^2^ DASH: from “*Drosophila*, *Arabidopsis*, *Synechocystis*, human”. ^3^ RING: from “Really Interesting New Gene”. ^4^ NTG: N-methyl-N′-nitro-N-nitrosoguanidine.

## Data Availability

Data are contained within the article and Supplementary Materials.

## Supplementary Materials

The following supporting information can be downloaded at: https://www.mdpi.com/article/10.3390/jof10030203/s1. Table S1. Sequences of primers used for sequencing *carS* mutant alleles. Supplementary Figure S1. Fusarin production and *fus1* expression by the wild type and *carS* mutants SF134, grown under different conditions of nitrogen and illumination.
