# Supplementary material for: Three Genes Involved in Different Signaling Pathways, carS, wcoA, and acyA, Participate in the Regulation of Fusarin Biosynthesis in Fusarium fujikuroi
Source: J Fungi (Basel). 2024 Mar 8;10(3):203. doi: 10.3390/jof10030203 (PMC10971159; doi:10.3390/jof10030203)
Supplement: Supplementary file 1 [file jof-10-00203-s001.zip › jof-2853874-supplementary.pdf]

Three genes involved in different signaling pathways, *carS*, *wcoA* and *acyA*, participate in the regulation of fusarin biosynthesis in *Fusarium fujikuroi*

Violeta Díaz-Sánchez, Marta Castrillo, Jorge García-Martínez, Javier Avalos, and M. Carmen Limón

SUPPLEMENTARY MATERIAL

Table S1. Sequences of primers used for sequencing *carS* mutant alleles

Name	Sequence
Foxy 1F	5'-CGCACGCAATCTATAGACGT-3'
Foxy 1R	5'-ATCATCAGCATCAGGAGGTG-3'
Foxy 2F	5'-GAGGCCGATATATGCTACGA-3'
Foxy 2R	5'-TCCTCGAGGCTAACATCGTC-3'
Foxy 3F	5'-CCTTGACGGATACATCGTCG-3'
Foxy 3R	5'-CGAGAGATAGTAGGGCAAGC-3'
Foxy 4F	5'-CTGGTGTATGACGATCTCTA-3'
Foxy 4R	5'-AGGTTCGTGATCGGAATCGG-3'

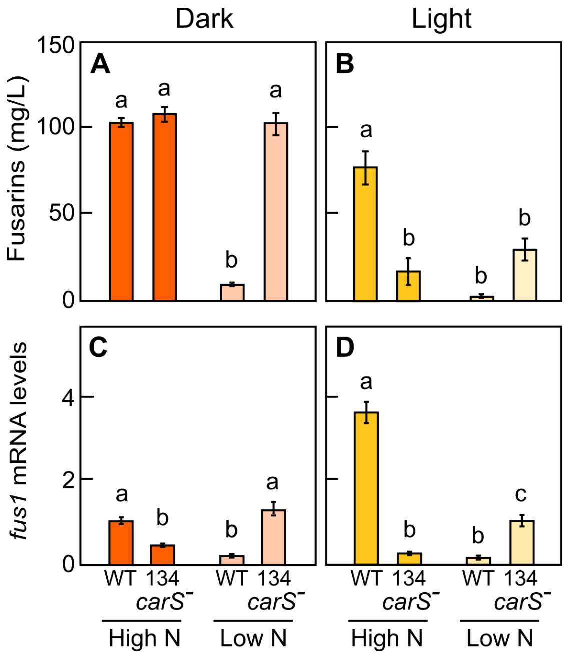

Supplementary Figure S1. Fusarin production and *fus1* expression by the wild type and *carS* mutants SF134, grown under different conditions of nitrogen and illumination. (**A-B**) Fusarins in the filtrates of 7-day-old cultures at 30 °C in minimal medium with Asn. (**A**): Cultures with high and low N concentration (20 mM asparagine) in the dark; **(B):** Cultures with low-N and high concentration (4.2 mM asparagine) under illumination. Data show mean and standard deviations from two independent experiments; (**C-D**) Relative *fus1* mRNA amounts in the mycelia from the cultures whose fusarin production is shown in the upper graphs. Data represent mean and standard deviation from 4 measurements from 2 independent experiments. High N: 20 mM asparagine. Low N: 4.2 mM asparagine. Statistically significant differences are indicated with different letters according to Tukey HSD test for a significant level of $\alpha = 0.05$.
